# Key residues of the receptor binding motif in the spike protein of SARS-CoV-2 that interact with ACE2 and neutralizing antibodies

**DOI:** 10.1038/s41423-020-0458-z

**Published:** 2020-05-15

**Authors:** Chunyan Yi, Xiaoyu Sun, Jing Ye, Longfei Ding, Meiqin Liu, Zhuo Yang, Xiao Lu, Yaguang Zhang, Liyang Ma, Wangpeng Gu, Aidong Qu, Jianqing Xu, Zhengli Shi, Zhiyang Ling, Bing Sun

**Affiliations:** 1 State Key Laboratory of Cell Biology, Shanghai Institute of Biochemistry and Cell Biology, Center for Excellence in Molecular Cell Science, Chinese Academy of Sciences, 200031 Shanghai, China; 2 School of Life Science and Technology, ShanghaiTech University, 201210 Shanghai, China; 3 Shanghai Public Health Clinical Center, Fudan University, 201508 Shanghai, China; 4 Key Laboratory of Special Pathogens, Wuhan Institute of Virology, Center for Biosafety Mega-Science, Chinese Academy of Sciences, 430071 Wuhan, China; 5 Shanghai Institute of Biological Products, 200052 Shanghai, China

**Keywords:** SARS-CoV-2, spike protein, receptor binding motif, cross-neutralizing antibody, substitution mutation, Immunization, Humoral immunity, Infection

## Abstract

Coronavirus disease 2019 (COVID-19), caused by the novel human coronavirus SARS-CoV-2, is currently a major threat to public health worldwide. The viral spike protein binds the host receptor angiotensin-converting enzyme 2 (ACE2) via the receptor-binding domain (RBD), and thus is believed to be a major target to block viral entry. Both SARS-CoV-2 and SARS-CoV share this mechanism. Here we functionally analyzed the key amino acid residues located within receptor binding motif of RBD that may interact with human ACE2 and available neutralizing antibodies. The in vivo experiments showed that immunization with either the SARS-CoV RBD or SARS-CoV-2 RBD was able to induce strong clade-specific neutralizing antibodies in mice; however, the cross-neutralizing activity was much weaker, indicating that there are distinct antigenic features in the RBDs of the two viruses. This finding was confirmed with the available neutralizing monoclonal antibodies against SARS-CoV or SARS-CoV-2. It is worth noting that a newly developed SARS-CoV-2 human antibody, HA001, was able to neutralize SARS-CoV-2, but failed to recognize SARS-CoV. Moreover, the potential epitope residues of HA001 were identified as A475 and F486 in the SARS-CoV-2 RBD, representing new binding sites for neutralizing antibodies. Overall, our study has revealed the presence of different key epitopes between SARS-CoV and SARS-CoV-2, which indicates the necessity to develop new prophylactic vaccine and antibody drugs for specific control of the COVID-19 pandemic although the available agents obtained from the SARS-CoV study are unneglectable.

## Introduction

Coronavirus disease 2019 (COVID-19) is a respiratory tract infection caused by a newly emergent coronavirus, SARS-CoV-2, which was first recognized in December 2019. Globally, as of 2:00 a.m. CEST, 25 April 2020, there have been 2,724,809 confirmed cases of COVID-19, including 187,847 deaths, reported to World Health Organization (https://covid19.who.int/). Genetic sequencing of the virus suggests that SARS-CoV-2 is a betacoronavirus closely linked to SARS-CoV.^1,2^ The research on SARS-CoV provided useful information that may be directly used in the battle against SARS-CoV-2, but the novel coronavirus also has different characteristics in some respects, which need more in-depth study.

Many groups have shown that SARS-CoV-2 utilizes the homotrimeric spike (S) glycoprotein to bind to the functional receptor human ACE2 (hACE2); this mechanism for viral entry is also used by SARS-CoV.^3,4^ The RBD in the S protein mediates the binding of the virus to host cells, which is a critical step for the virus to enter target cells. According to the high-resolution crystal structure information acquired thus far,^5–7^ the receptor-binding motif (RBM) is the main functional motif in RBD and is composed of two regions (region 1 and region 2) that form the interface between the S protein and hACE2.^8^ The region outside the RBM also plays an important role in maintaining the structural stability of the RBD.^9^

According to amino acid alignment studies, the sequence identity of the RBD shared by SARS-CoV and SARS-CoV-2 is 73.5%.^10^ However, the identity of RBM, the most variable region of RBD, is only 47.8%. Although the identity of the amino acids in the RBM region is low, the binding mechanism is similar for the two viruses.^5–7,11–13^ The conservation of amino acid sequences suggests that the RBDs of the two viruses may elicit cross-reactive antibodies which may have the potential for cross protection. It is currently unclear whether the variable RBMs of the two viruses can induce cross-reactive antibodies.

In this study, we compared the SARS-CoV-2 and SARS-CoV RBD affinity for hACE2 and explored the possibility of cross protection by antibodies targeting these RBDs. By creating single amino acid substitution mutations in the SARS-CoV and SARS-CoV-2 RBM sequences, we demonstrated that SARS-CoV-2 has two types of amino acid residues to maintain its binding activity with hACE2: receptor binding was enhanced by introducing amino acid changes at P499, Q493, F486, A475 and L455, and receptor binding was diminished by replacing residues N501, Q498, E484, T470, K452 and R439. An animal immunization study revealed that the RBDs of SARS-CoV and SARS-CoV-2 are potential antigens that induce strong clade-specific neutralizing antibodies in mice, while the cross-neutralizing effect is much weaker. This finding was due to the differences in antigenicity of the RBDs in the 2 viruses, which was carefully verified with the available neutralizing monoclonal antibodies(mAbs) against SARS-CoV and/or SARS-CoV-2. Finally, the potential epitope of HA001, a newly developed SARS-CoV-2 receptor-blocking human antibody, was found to involve amino acids A475 and F486 in the SARS-CoV-2 RBD, which are newly discovered binding sites for neutralizing antibodies.

Overall, our study has revealed the presence of different key epitopes between SARS-CoV and SARS-CoV-2, which indicates the necessity to develop new prophylactic vaccine and antibody drugs for specific control of the COVID-19 pandemic although the available agents obtained from the SARS-CoV study are unneglectable.

## Materials and methods

### Cells, plasmids and antibodies

HEK293T cells (ATCC CRL-3216) were cultured at 37°C with 5% CO_2_ in Dulbecco’s modified Eagle’s medium (DMEM; Invitrogen) supplemented with 100 U penicillin per ml, 100 μg streptomycin per ml, and 10% fetal calf serum. ExpiCHO-S mammalian cells (Invitrogen) were cultured in ExpiCHO™ Expression Medium (invitrogen), supplemented with 1% penicillin-streptomycin at 37°C with 8% CO_2_. The 80R, m396, S230, CR3022 and N-176-15 VH and VL sequences were synthesized (GenScript) and cloned into human IgG1 scaffold. HA001 targeting SARS-CoV-2 RBD that was generated by phage display was provided by Shanghai Sanyou Biopharmaceuticals. The expressing plasmids encoding full length of SARS-CoV S protein, SARS-CoV-2 S protein and human ACE2 were purchased from Sino Biological. The RBD domain encompassing residues 306-527 of SARS-CoV S protein, residues 318-541 of SARS-CoV-2 S protein were cloned into the pcDNA3.1 mammalian expression vector by incorporating an immunoglobulin (Ig) heavy chain (H) signal peptide at the N-terminus and a human IgG1 Fc tag at the C-terminus.

### Single amino acid substitution mutagenesis of the RBDs

The DNA sequences encoding the RBD of SARS-CoV or SARS-CoV-2 were fused in frame with an N-terminal human IgE signal peptide and a C-terminal 6 His tag and cloned into a pBudCE4.1 vector (Invitrogen). The residues selected for mutagenesis were based on an amino acid sequence alignment and the structural information of the RBD-ACE2 binding interface. Single amino acid substitution mutagenesis was induced with a commercialized KOD-Plus mutagenesis kit (TOYOBO). All the mutations were verified by DNA sequence analysis (Biosune). To express the wild-type and mutant RBDs, ExpiCHO-S cells were plated in six-well plates and transiently transfected with these plasmids. The supernatants were harvested 96 h after transfection.

### Expression and purification of the RBD-hFc and mAbs

The mAbs and RBD-hFc were produced by transient transfection of ExpiCHO-S cells (Invitrogen). The supernatants from the RBD-hFc transfected cells were collected after 4 days and from the mAb-transfected cells after 7 days. The supernatants were affinity purified by protein G chromatography (GE Healthcare) and dialysed against PBS overnight at 4 °C.

### Biolayer interferometry analysis of the SARS-CoV and SARS-CoV-2 RBD binding affinity for hACE2

Biolayer interferometry was performed using an Octet Red96 instrument (ForteBio, Inc.). A 5 μg/ml concentration of SARS-CoV and SARS-CoV-2 RBD-hFc was immobilized on an anti-human IgG-Fc (AHC)-coated biosensor surface for 300 s. The baseline interference phase was obtained by measurements taken for 60 s in kinetics buffer (KB: 1x PBS and 0.02% Tween-20), and then, the sensors were subjected to association phase immersion for 400 s in wells containing recombinant hACE2 diluted in KB. Then, the sensors were immersed in KB for as long as 400 s in the dissociation step. The mean Kon, Koff and apparent *K*_D_ values of the SARS-CoV and SARS-CoV-2 RBD binding affinities for ACE2 were calculated from all the binding curves based on their global fit to a 1:1 Langmuir binding model with an R2 value of ≥0.95.

### Pseudo-typed virus infection assay

SARS-CoV and SARS-CoV-2 pseudo-typed viruses were produced as previously described.^14^ Briefly, plasmids coding full-length S protein and pNL4-3.luc.RE were cotransfected into 293T cells in 10 cm dishes. The supernatants were harvested 48 h after transfection and diluted in complete DMEM mixed with or without an equal volume (50 μl) of diluted serum or antibody and then incubated at 37 °C for 1 h. The mixtures were transferred to HEK 293 T cells stably expressing human ACE2. The cells were incubated at 37 °C for 48 h, lysed with passive lysis buffer and tested for luciferase activity (Promega USA). The percent neutralization was calculated by comparing the luciferase value of the antibody or serum group to that of the virus-only control.

### Syncytia formation assay

In brief, HEK293T cells were transfected (when ~60–70% confluent in six-well plates) by Lipofectamine 2000 with plasmids encoding a codon-optimized full-length form of the SARS-CoV and SARS-CoV-2 S protein or a control plasmid. In parallel, another group of HEK293T cells was transfected with plasmids encoding hACE2. Twenty-four hours after transfection, the two groups of cells were trypsinized and mixed at a 1:1 ratio and then plated on 24-well plates. After 48 h of coculturing, multinucleated giant cells were observed. Images were collected and analysed with an Olympus IX53 confocal microscope.^15^

### Animal immunization

Groups of five age- and weight-matched male C57BL/6 mice were immunized intramuscularly with 25 μg recombinant SARS-CoV- RBD hFc, SARS-CoV-2 RBD hFc or hIgG, as a control, in the presence of adjuvant QuickAntibody (BioDragon). A booster was subsequently administered at 3-week intervals in all cases. The animals were sacrificed 14 days after the second immunization, and serum was collected. In terms of neutralization assays, the serum samples were heat inactivated at 56 °C for 30 min.

### Enzyme-linked immunosorbent assay (ELISA)

To confirm whether the antibodies recognized SARS-CoV RBD or SARS-CoV-2 RBD, 96-well microwell plates (Nunc) were coated with 50 ng/well recombinant SARS-CoV RBD hFc and SARS-CoV-2 RBD hFc in 0.1 M sodium carbonate-bicarbonate buffer (pH 9.6) and incubated overnight at 4 °C. After blocking at 37 °C for 2 h with ovine serum albumin (2%) in PBS, the ELISA plates were washed, and diluted antibodies were added for a 2-h incubation. HRP-conjugated goat anti-human Fc or HRP-conjugated goat anti-mouse Fc antibody (Sigma) was used to detect the bound antibodies.

To determine the residues that contributed to the binding of the SARS-CoV and SARS-CoV-2 RBDs to ACE2 or neutralizing antibodies. The concentration of RBD mutants in the culture supernatant was measured by sandwich enzyme-linked immunosorbent assay. Specifically, CR3022 and R007 (Sino Biological), which cross-react with both the SARS-CoV and SARS-CoV-2 RBD, were used to coat plates, and cell supernatant diluted 50-fold or 500-fold was then added and treated with the two mAbs. Serially diluted purified SARS-CoV and SARS-CoV-2 RBD (2-fold dilution of initial 200 ng/ml) were used as standards. Subsequently, the bound antigen was detected by an HRP-conjugated mouse anti-His mAb. The concentration of the RBD mutants was determined according to the standard curve. Then, another ELISA was performed to analyse the relative binding activity of these RBD mutants for ACE2 and the mAbs. The RBD mutants (100 and 200 ng/ml) were incubated on plates pre-coated with 500 ng mAbs or ACE2. After 2 h of incubation at 37 °C, the binding affinity of the RBD mutants for mAbs or ACE2 was detected by an HRP-conjugated mouse anti-His mAb. The binding signals of the mutants to the mAbs were compared to those of the wild-type virus proteins.

### Receptor blocking assay

To investigate the ability of the mAbs and sera from immunized mice to block SARS-CoV-2 spike protein binding to ACE2, serially diluted mAbs (3-fold dilution of initial 30 µg/ml) and sera (3-fold dilution of initial 1:10) were added to plates pre-coated with 100 ng/well of the recombinant SARS-CoV RBD-his, SARS-CoV-2 RBD-his (Sino Biological) and incubated for 1 h at 37 °C. Then, 150 ng/ml of the biotin-labelled recombinant hACE2-his (Novoprotein) expressed by 293 T cells was added to the plates. After 2 h of incubation at 37 °C, the wells were washed and detected with HRP-conjugated streptavidin (R&D Systems). The plates were incubated for 1 h, followed by the addition of TMB substrate. The percentage of receptors blocked was determined by the reduced percentage of S binding to ACE2 as determined by comparison with the percentage obtained in the absence of serum. The fifty percent inhibitory concentration [IC_50_ (micrograms per millilitre)] was used as the inhibition value.

### Structure analysis

Local minimization was carried out by Prime after mutating Q498 to Y to simulate a conformation change within 5 Å in the amino acids around Q498 (PDB ID: 6LZG). The minimization shows that the aromatic ring of Y498 can form π-π stacking interactions with Y41 in hACE2, which enhances the RBS binding with hACE2. Structural figures were generated using PyMOL (The PyMOL Molecular Graphics System, Version 2.0 Schrödinger, LLC).

### Statistical analyses

All statistical analyses were performed using Graph Pad Prism 6 software. The *P* values shown in the figures and figure legends were determined using unpaired two-tailed Student’s *t*-tests (*P < 0.05, **P < 0.01, and ***P < 0.001; not significant (NS)).

## Results

### Both the SARS-CoV and SARS-CoV-2 RBDs bind to hACE2 for virus entry

**Fig. 1:**
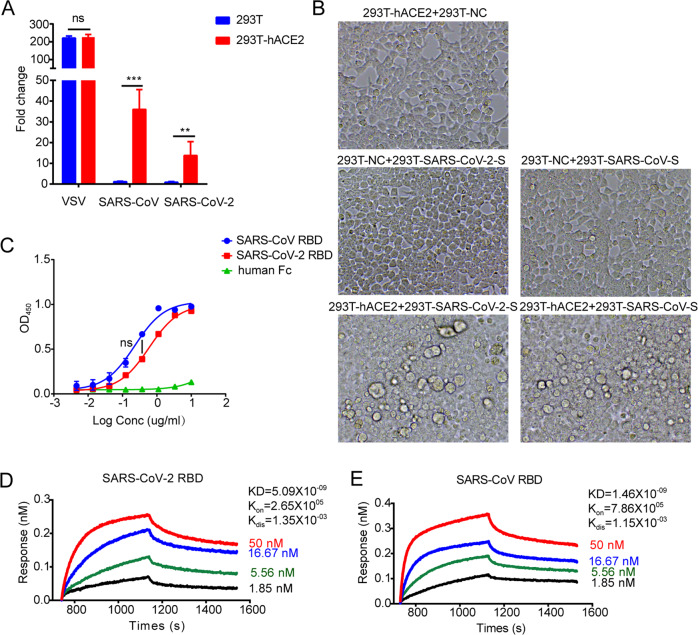
Both the SARS-CoV-2 RBD and SARS-CoV RBD bind to hACE2. **a** Receptor-dependent infection of SARS-CoV-2 and SARS-CoV pseudo-typed virus entry into hACE2^+^ 293 T cells. 293T cells stably expressing hACE2 were infected with SARS-CoV-2 or SARS-CoV pseudo-typed viruses, and the cells were harvested to detect the luciferase activity. Fold changes were calculated by comparison to the levels in the uninfected cells. VSV pseudo-typed viruses were included as controls. **b** Syncytia formation between S protein- and hACE2-expressing cells. 293T cells transfected with hACE2 plasmid were mixed at a 1:1 ratio with 293T cells transfected with plasmid encoding S protein from SARS-CoV-2 (bottom left) or SARS-CoV (bottom right). As controls, 293T cells transfected with an empty plasmid were either mixed at a 1:1 ratio with 293T cells transfected with the hACE2 plasmid (top row), S protein from SARS-CoV-2 (middle left) or SARS-CoV (middle right). Images were photographed at ×20 magnification. Representative images are shown. **c** Dose-dependent binding of the SARS-CoV-2 RBD to soluble hACE2 as determined by ELISA. The binding of both the SARS-CoV-2 RBD and SARS-CoV RBD with an Fc tag on hACE2 was tested. Human Fc was included as a control. Data are presented as the mean OD450 ± s.e.m. (*n* = 2). **d** Binding profiles of the SARS-CoV-2 RBD and SARS-CoV RBD to the soluble hACE2 receptor measured by biolayer interferometry in an Octet RED96 instrument. The biotin-conjugated hACE2 protein was captured by streptavidin that was immobilized on a chip and tested for binding with gradient concentrations of the soluble RBD of S proteins from SARS CoV and SARS CoV-2. Binding kinetics were evaluated using a 1:1 Langmuir binding model by ForteBio Data Analysis 9.0 software

To confirm that the infectivity of SARS-CoV and SARS-CoV-2 is dependent on hACE2, we constructed pseudo-typed SARS-CoV and SARS-CoV-2 by the co-transfection of a plasmid encoding Env-defective luciferase-expressing HIV-1 (pNL4-3.luc.RE) and a plasmid expressing the full-length S protein of SARS-CoV or SARS-CoV-2 into HEK293T cells. The HEK293T cells expressing or not expressing hACE2 were treated with pseudo-typed virus-containing supernatants. The pseudo-typed SARS-CoV and SARS CoV-2 showed much higher infectivity in the HEK293T cells expressing hACE2 than they did in the HEK293T cells not expressing hACE2, while there was no significant difference in the pseudo-typed VSVG infectivity in the HEK293T cells with or without hACE2 (Fig. 1a). The results indicated that hACE2 is a receptor used by both SARS-CoV and SARS-CoV-2 to enter the cells. Because syncytial formation has been observed in cultured Vero E6 cells infected with SARS-CoV,^16^ we also sought to determine whether HEK293T cells expressing the SARS-CoV-2 S protein could fuse with HEK293T cells expressing hACE2. As expected, the HEK293T cells transfected with hACE2 formed many syncytia with cells expressing the SARS-CoV S protein. In contrast, for the 293T cells expressing hACE2, the S protein of SARS-CoV or SARS-CoV-2 alone did not form syncytia. The HEK293T cells expressing the S protein of SARS-CoV-2 also efficiently formed syncytia with hACE2-transfected cells (Fig. 1b). As the RBD is the key region for SARS-CoV S-hACE2 recognition, we investigated the binding affinity of hACE2 and S protein though biolayer interferometry (BLI) and enzyme-linked immunosorbent assay (ELISA). The biotin-conjugated hACE2 protein was captured by streptavidin that was immobilized on a chip and tested for binding with gradient concentrations of soluble RBD from SARS-CoV and SARS-CoV-2. The equilibrium dissociation constant (KD) of SARS-CoV-2-RBD binding to hACE2 was calculated to be 5.09 nM, which is comparable to that of the SARS-RBD: 1.46 nM^6^ (Fig. 1d). Similar data were obtained through ELISAs (Fig. 1c). Taken together, these results confirmed that both SARS-CoV and SARS-CoV-2 utilize the RBD to bind to hACE2 for virus entry.

### Distinct immunogenicity of the SARS-CoV RBD and SARS-CoV-2 RBD

**Fig. 2:**
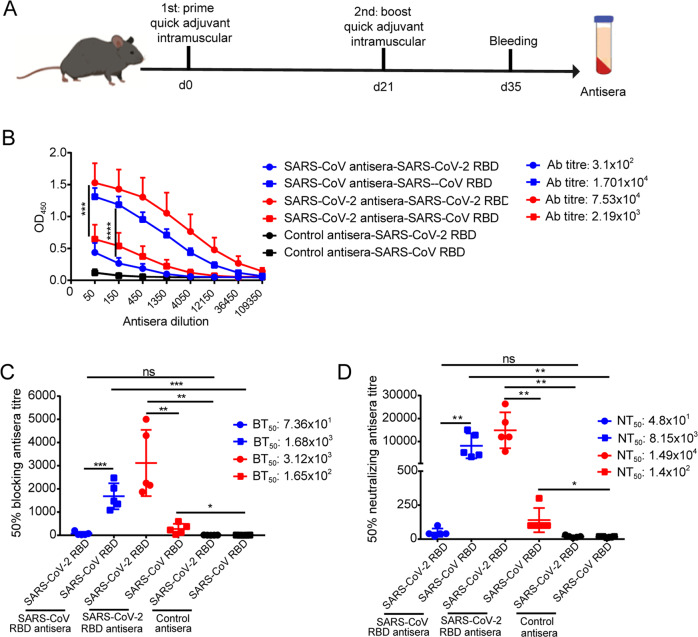
The antibody response induced by recombinant RBD of SARS-CoV and SARS-CoV-2 in mice. **a** Schematic of the vaccine regimen. Five C57BL/6 mice per group were immunized two times (2–3 weeks apart) intramuscularly with 25 µg of the SARS CoV-2 RBD-hFc or SARS CoV RBD-hFc protein in combination with quick adjuvant. Mice immunized without the RBD protein but with hIgG were included as controls. Mice were sacrificed on day 35 after immunization, and antisera were collected for subsequent tests. **b** Cross-reactivity of SARS-CoV-2-RBD- or SARS-CoV-RBD-specific mouse sera against the SARS-CoV RBD or SARS-CoV-2 RBD as determined by ELISA. Mouse antisera were serially diluted three-fold and tested for binding to the SARS-CoV RBD or SARS-CoV-2 RBD. The IgG antibody (Ab) titres of SARS-CoV-2 antisera (red), SARS-CoV antisera (blue) and control antisera (black) were calculated at the endpoint dilution that remained positively detectable for the SARS-CoV-2 RBD or SARS-CoV RBD. The data are presented as the mean A450 ± s.e.m. (*n* = 5). **c** Cross-competition of SARS-CoV-2-RBD- or SARS-CoV-RBD-specific mouse sera and hACE2 with the SARS-CoV RBD or SARS-CoV-2 RBD as determined by ELISA. The data are presented as the mean blocking (%) ± s.e.m. (*n* = 5). Fifty percent blocking antibody titres (BT_50_) against the SARS-CoV pseudo-typed virus or SARS-CoV pseudo-typed virus were calculated. **d** Cross-neutralization of SARS-CoV-2-RBD- or SARS-CoV-RBD-specific mouse sera against SARS-CoV-2 or SARS-CoV pseudo-typed virus entry, measured by pseudo-typed virus neutralization assay. The data are presented as the mean neutralization (%) ± s.e.m. (*n* = 5). Fifty percent neutralizing antibody titres (NT_50_) against the SARS-CoV-2 or SARS-CoV pseudo-typed virus were calculated

Since sequence alignment indicated high conservation of the SARS-CoV and SARS-CoV-2 RBDs with a shared identity of 76%, we sought to determine whether the RBDs induced cross-reactive immune responses to confer protection from both viruses. To address this question, we conducted an in vivo immunization experiment to determine the cross-reactivity of the antibodies induced by the SARS-CoV RBD and SARS-CoV-2 RBD (Fig. 2a). First, we performed ELISAs to detect the cross-reactivity of the sera from mice immunized with recombinant SARS-CoV-2 RBD or SARS-CoV RBD. The results showed that the SARS-CoV RBD antiserum reacted strongly to the SARS-CoV RBD, with a mean antibody titre of 1.701 × 10^4^ and with a lower titre upon SARS-CoV-2 RBD exposure (mean antibody titre: 3.1 × 10^2^). Similarly, the SARS-CoV-2 RBD antiserum reacted strongly to the SARS-CoV-2 RBD, with a high antibody titre (7.53 × 10^4^) and with a lower titre upon SARS-CoV RBD exposure (2.19 × 10^3^) (Fig. 2b). Correspondingly, we investigated the efficiency of the antiserum cross-blocking of the interaction between hACE2 and the S protein RBD. The results revealed that the SARS-CoV RBD antiserum strongly blocked the interaction between hACE2 and the SARS-CoV RBD with a mean 50% blocking antiserum titre (BT_50_: 1.68 × 10^3^) but very low BT_50_ upon exposure to the SARS-CoV-2 RBD (BT_50_: 73.6). Similarly, the SARS-CoV-2 RBD antiserum had a higher 50% blocking antiserum titre (BT_50_: 3.12 × 10^3^) for the SARS-CoV-2 RBD and hACE2 interaction but much lower cross-blocking efficiency for the SARS-CoV RBD and hACE2 interaction (BT_50_: 1.65 × 10^2^) (Fig. 2c). To confirm this observation, a pseudo-typed virus neutralization assay was performed to evaluate the cross-neutralization efficacy of the SARS-CoV RBD- or SARS-CoV-2 RBD-immunized mouse antiserum. In agreement with the results from the blocking assay, the neutralization activity of the mouse antisera was near completely clade-specific with very low cross-neutralization levels. The 50% neutralization antiserum titre (NT_50_) of the SARS-CoV-2 antiserum to the SARS-CoV-2 pseudo-typed virus was calculated to be 1.49 × 10^4^, and that of the SARS-CoV antiserum to the SARS-CoV pseudo-typed virus was 8.15 × 10^3^ (Fig. 2d). Therefore, these results suggest that there is distinct immunogenicity in the SARS-CoV RBD and SARS-CoV-2 RBD, explaining the limited amount of cross-protecting antibodies produced by the two viruses.

### Identification of key residues in the SARS-CoV and SARS-CoV-2 RBDs to determine receptor-binding levels

**Fig. 3:**
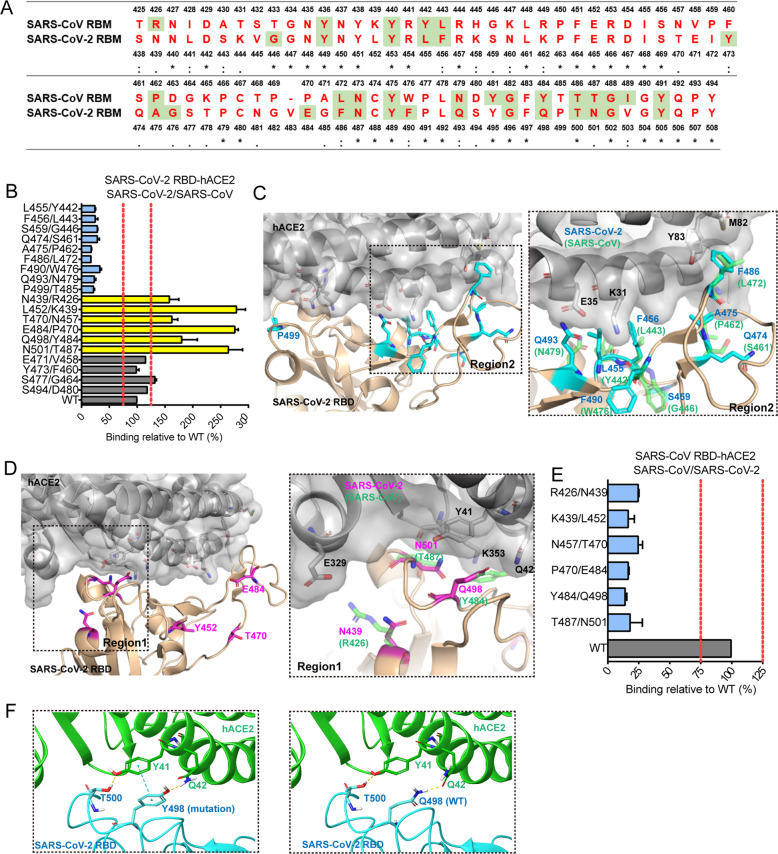
Single amino acid substitution mutagenesis of the SARS-CoV-2-RBD and SARS-CoV-RBD. **a** Sequence differences in the SARS-CoV and SARS-CoV-2 RBDs. RBM is in red. Previously, identified critical ACE2-binding residues are shaded in green. The conserved residues are marked with asterisks (*), the residues with similar properties between groups are marked with the colon symbol (:) and the residues with marginally similar properties are marked with the period symbol (.). **b** ACE2 binding with reciprocal amino acid substitutions in the SARS-CoV-2 RBD. Each value is calculated as the binding relative to that of the WT (%). The mean±S.E.M. of duplicate wells is shown for two independent experiments. The two red dotted lines represent 75% and 125% relative to the WT data, respectively. **c**, **d** Structural alignment of SARS-CoV-2-RBD and SARS-CoV-RBD binding with ACE2. The SARS-CoV-RBD complex (PDB ID: 2AJF) is superimposed on the SARS-CoV-2 RBD (PDB ID: 6lzj. grey: ACE2, wheat: SARS-CoV-2. Mutants that weaken the SARS-CoV-2 RBD binding with ACE2 are highlighted in cyan (**c**). The corresponding residues from SARS-CoV are indicated in green and are illustrated in detail (**c** left). Mutants that enhance ACE2 binding are highlighted in magenta (**d**). **e** ACE2 binding with reciprocal amino acid substitutions in the SARS-CoV RBD. Each value is calculated as the binding relative to that of the WT (%). The mean ± S.E.M. of duplicate wells is shown in two independent experiments. The two red dotted lines represent 75 and 125% relative to the WT data, respectively. **f** Molecular docking of the SARS-CoV 2 RBD carrying the Q498Y mutant in complex with hACE2. Q498Y formed π-π stacking with Y41 in hACE2: left, Y498; right, Q498

It is well known that the interaction of the interface between S-RBD and ACE2 plays a crucial role in their binding activity, with the RBM regions considered to be particularly important.^5^ However, the amino acids of the RBM were quite different, with a shared identity of only 47.8%. It was not clear which mutated residues within the RBM would alter its affinity for hACE2; thus, a functional analysis was needed. Based on the sequence alignment (Fig. 3a), 19 residues were selected, and single amino acid substitutions were added to mutate the SARS-CoV-2 and SARS-CoV RBM regions. The results showed that, in some mutants, the binding affinity of the SARS-CoV-2 RBD with hACE2 were decreased, but for others, it was increased or not affected (Fig. 3b). According to the high-resolution crystal structure information acquired thus far,^5–7,11,12^ the receptor-binding motif (RBM) is composed of two regions (region 1 and region 2) that form the interface of the S protein with hACE2, and we focused on the residues within either region 1 or region 2 (Fig. 3c).^17^ Interestingly, after 9 amino acid residues were mutated in the SARS-CoV-2 to SARS-CoV (L455/Y442, F456/L443, S459/G46, Q474/S461, A475/P462, F486/L472, F490/W476, Q493/N479 and P499/T485), their binding affinity for hACE2 was abolished, in contrast to that of the WT viruses (Fig. 3b, c), indicating that these residues are very important for the binding of SARS-CoV-2 to hAEC2. It is worth noting that 6 of the 9 residues, L455, F456, A475, F486, F490 and Q493, have been previously reported to be SARS-CoV-2 RBD-hACE2 interacting residues based on structure analysis.^5–7^ According to structure analysis, L455 and Q493 of the SARS-CoV-2 RBD have favourable interactions with hACE2 K31 and E35; upon binding, the salt bridge between the two hACE2 residues breaks, and each of the residues forms a hydrogen bond with Q493 in the SARS-CoV-2 RBM, thus enhancing the binding to hACE2^6^ (Fig. 3c), a finding in agreement with our mutagenesis results (Fig. 3b). The introduction of F486 in the SARS-CoV-2 RBD enhanced the hACE2 binding affinity by creating a hydrophobic pocket involving M82 and Y83 in hACE2^6^ (Fig. 3c). In addition, we report here, for the first time, that the other three SARS-CoV-2 substitution mutants, S459/G443, Q474/S461 and P499/T485, which do not directly contact hACE2, also reduce the binding affinity. These three SARS-CoV-2 residues may strengthen the structure and stabilize the hACE2-SARS-CoV-2 binding interface.

In contrast, we also identified 6 substitution mutants in the SARS-CoV-2 RBD, N439/R426, L452/K439, T470/N457, E484/P470, Q498/Y484 and N501/T487, that enhanced the binding affinity, which provides clues for monitoring the increased infectibility of natural RBD mutations during the transmission of the virus (Fig. 3b, d). We speculate that these residues may be critical for SARS-CoV RBD binding to hACE2. As expected, for SARS-CoV, when R426, K439, N457, P470, Y484 and T487 were replaced with the corresponding residues in the SARS-CoV-2 RBD, the binding activity of the SARS-CoV RBD was dramatically decreased compared with that of the RBD in the WT virus (Fig. 3e). Previous structural studies identified R426, Y484 and T487 as key residues for the SARS-CoV RBD binding to hACE2,^4,18^ which was confirmed based on the data provided above (Fig. 3d). According to the structure analysis, the replacement of SARS-CoV-2 RBD N439 with R426 increased the hACE2 binding affinity by introducing a strong salt bridge between R426 in the SARS-CoV-2 RBD and E329 in hACE2 (Fig. 3d). Molecular docking showed that the substitution of SARS-CoV-2 RBD Q498 with Y484 formed π-π stacking interactions with Y41 on hACE2, although hydrogen bonds were also involved, which explains the enhanced hACE2 binding (Fig. 3f). Moreover, according to the structural data, both SARS-CoV-2 RBD N501 and SARS-CoV RBD T487 have similar interactions with Y41 and K353 in hACE2, but the replacement of SARS-CoV-2 RBD N501 with T487 significantly enhanced its binding with ACE2. The enhanced binding activity of the SARS-CoV-2 RBD mutant N501/T487 may be due to the increased support provided by this residue to stabilize the overall structure of RBD or strengthen the network of hydrophilic interactions (Fig. 3d). These results suggest that some residues critical for SARS-CoV RBD-ACE2 recognition, namely, R426, K439, N457, P470, Y484 and T487, were different at the corresponding positions in SARS-CoV-2. In contrast, the key residues for SARS-CoV-2 recognition, L455, A475, F486 and Q493, were also different at the corresponding positions in SARS-CoV-2.

In summary, the overall receptor-binding mode of the SARS-CoV-2 and SAR-CoV RBDs was quite similar, but the detailed interaction patterns were substantially different, which might explain the distinct of immunogenic features of the SARS-CoV-2 and SAR-CoV RBDs, which induce the production of clade-specific neutralizing Abs.

### A panel of mAbs revealed limited cross-neutralization in SARS-CoV-2 and SARS-CoV

**Fig. 4:**
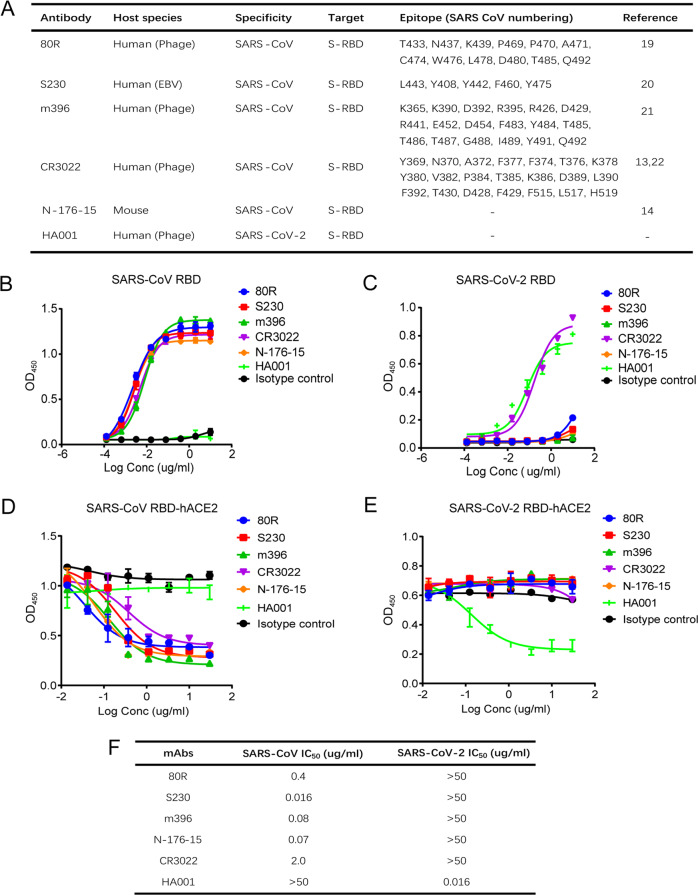
Cross-reactivity of the RBD-targeting neutralizing mAbs against SARS-CoV and SARS-CoV-2. **a** Characteristics of the neutralizing mAbs against the SARS CoV-2 RBD and SARS CoV RBD. **b**, **c** Dose-dependent binding of SARS-CoV and SARS-CoV-2 mAbs to the SARS-CoV RBD (**b**) or SARS-CoV-2 RBD (**c**) as determined by ELISA. Isotype antibody was included as a control. Data are presented as the mean OD450 ± s.e.m. (*n* = 2). **d**, **e** Dose-dependent competition of the SARS-CoV-2 or SARS-CoV mAbs and hACE2 with the SARS-CoV RBD (**d**) or SARS-CoV-2 RBD (**e**) as measured by ELISA. Data are presented as the mean OD450 ± s.e.m. (*n* = 2). **f** IC_50_ values were determined for a panel of mAbs neutralizing the SARS-CoV-2 or SARS-CoV pseudo-typed viruses. Representative data are shown

Then, we investigated the immunogenic characteristics of the SARS-CoV-2 RBD and SARS-CoV RBD by using a panel of neutralizing mAbs against the SARS-CoV RBD, including those targeting 80R, S230, m396, CR3022 and N-176-15.^14,19–22^ We also tested the human mAb HA001 against the SARS-CoV-2 RBD (Fig. 4a). All the antibodies, except for CR3022, showed only clade-specific binding activity (Fig. 4b, c). All the neutralizing antibodies selected disrupted only clade-specific RBD-hACE2 interactions (Fig. 4b, c). Further pseudo-typed virus assays confirmed that all the SARS-CoV-RBD mAbs failed to neutralize SARS-CoV-2, and HA001 did not neutralize SARS-CoV. As expected, the SARS-CoV-RBD mAbs neutralized SARS-CoV with IC_50_ values ranging from 0.016 to 2.0 µg/ml, and HA001 neutralized SARS-CoV-2 with an IC_50_ value of 0.016 µg/ml (Fig. 4d). These data indicate that the tested mAbs targeting the SARS-CoV-2 RBD could not cross protect SARS-CoV, and vice versa, which may be due to the different sequence of the RBDs in the two viruses. This result emphasized the necessity of developing specific vaccines and antibodies against SARS-CoV-2 S.

### Substitute mutagenesis of the RBM to identify key residues for neutralizing antibody recognition

**Fig. 5:**
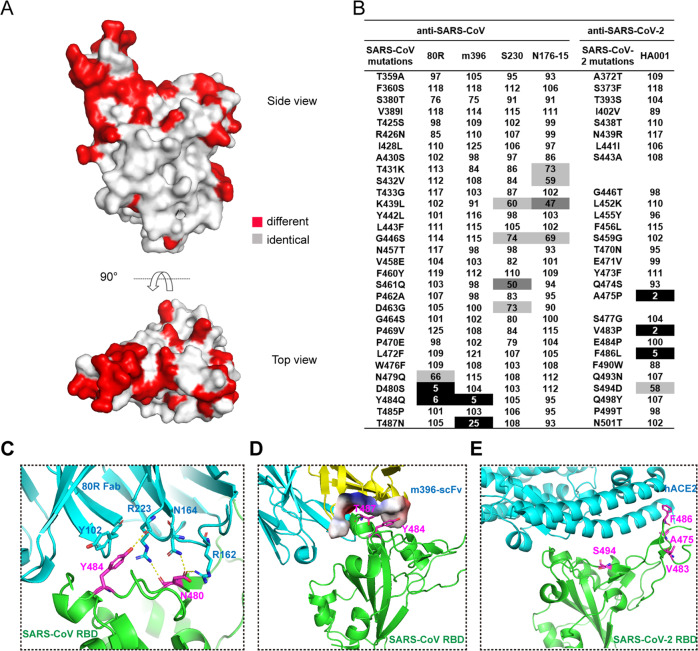
Recognition pattern of mAbs to single amino acid substitute mutants of SARS-CoV or SARS-CoV-2 RBD. **a** Sequence conservation in the SARS-CoV and SARS-CoV-2 RBDs in a surface representation. Red, different; grey, identical. **b** Site mutagenesis scanning. The SARS-CoV and SARS-CoV-2 RBD mutant panel includes the reported antibody epitope positions and sequence changes within the RBMs. Relative binding to the wild-type: 0–25% presented in black; 25–50%, presented in dark grey; 50–75% presented in light grey; >75%, presented in white. The results shown represent the mean percentage of binding signal for the mAbs bound to the mutants relative to that of the wild-type RBD in at least two independent experiments. **c** Interaction of Y484 and D480 in the SARS-CoV RBD with 80 R (PDB ID: 2ghw). Polar interactions are indicated by yellow dashed lines. **d** Interaction of Y484 and T487 in the SARS-CoV RBD with m396 (PDB ID: 2dd8). Yellow: heavy chain, cyan: light chain. The binding surface of m396 is shown by electrostatic surface representations. **e** The residues that are important for HA001 binding are on the interface of the ACE2 and RBD (PDB ID: 6VW1)

To evaluate the identified key residues for antibody recognition, a panel of neutralizing mAbs against the SARS-CoV RBD and one neutralizing mAb against the SARS-CoV-2 RBD (HA001) were selected (Fig. 4a). Each antibody showed only clade-specific binding activity (Fig. 4b). To investigate the key residues of the RBD in terms of the recognition of the clade-specific neutralizing antibodies, single amino acid substitution mutagenesis scanning, based on the reported antibody epitope positions and sequence changes within the RBM, was performed (Fig. 5a, b).

In our mutagenesis assays, four SARS-CoV-2 RBD mutants, A475/P462, V483/P469, F486/L472 and S494/D480, failed to bind HA001 (Fig. 5b). Among these four amino acids, A475 and F486 were critical for the binding activity of the RBD in interaction with HA001 and hACE2 (Fig. 5e). These observations demonstrate that HA001 neutralizes SARS-CoV-2 by competing for the same critical residues in the β 5 loop of the RBD and thus blocking receptor binding.^5^

We employed a similar strategy to test the binding affinity of SARS-CoV mAbs for SARS-CoV RBD mutants. The data showed that the binding of 80R was significantly suppressed when SARS-CoV RBD residues D480 and Y484 were mutated to S494 and Q498, the corresponding amino acids in the SARS-CoV-2 RBD, whereas other sequence changes had no effect (Fig. 5b). Based on the crystal structures of the SARS-CoV RBD-80R complex,^19^ D480 and Y484 were shown to play strategic roles in the interaction between 80R and SARS-CoV-RBD (Fig. 5c). Replacement of Y484 with Q weakened the interaction between SARS-CoV-RBD and 80R by eliminating the strong π-π stacking interactions of Y102 in the CDRH3 region of 80R.

Similarly, for m396, replacing the residues of SARS-CoV RBD Y484 and T487 with the Q498 and N501 residues in the SARS-CoV-2 RBD significantly reduced its binding to the SARS-CoV RBD, compared to that of the other mutants (Fig. 5b). Based on crystal structure analysis of the SARS-CoV RBD-m396 complexes, the m396 CDRH1 region made contact with the hydrophobic residues Y484, T486 and T487 of the SARS-CoV RBD. The replacement of SARS-CoV RBD Y484 with hydrophilic Q disrupted the hydrophobic interaction with m396. SARS-CoV RBD T487 inserted into a hydrophobic pocket involving m396 and the replacement of this residue with SARS-CoV-2 RBD N501 may have led to a change in the conformational structure of the hydrophobic pocket, thus weakening both SARS-CoV RBD-hACE2 binding and SARS-CoV RBD-m396 binding (Fig. 5d).

In brief, we identified A475 and F486 in the SARS-CoV-2 RBD and Y484 and T487 in the SARS-CoV RBD as the key residues for the recognition of both their common functional receptor hACE2 and neutralizing antibodies. Due to the different immunogenicity of the RBMs in the 2 viruses, the neutralizing antibodies failed to show cross-reactivity.

In addition, several mutations to the SARS-CoV RBD, which were mainly located in the hypervariable region A430-D463, moderately reduced its binding activity to S230 and N-176-15. All of the mutations may synergistically contribute to poor cross-reactivity by inducing conformational changes at the binding surface. We observed that K439 was important for both S230 and N176-15 binding to the SARS-CoV RBD, and it is also a key residue for the SARS-CoV RBD binding to hACE2.

In conclusion, the variations in the RBMs, especially those residues involved in ACE2 recognition, may be critical for the failure of the crossing neutralization of the antibodies targeting the RBDs.

## Discussion

The World Health Organization officially declared SARS-CoV-2 a pandemic on 11 March 2020. The pandemic has become increasingly serious worldwide. With the deepening of research to SARS-CoV-2 and COVID-19, the previous optimistic speculation has been gradually replaced by expectations for a long-term fight against the virus. To control pandemics, prophylactic vaccines and effective drugs are urgently required.

According to published articles, both SARS-CoV-2 and SARS-CoV utilize the same human receptor, ACE2, which was also confirmed in our study. Hence, the S protein, especially its RBD, which is responsible for hACE2 binding, is the most promising target for the development of SARS-CoV vaccines and antibody-based drugs.^23^ Based on the newly disclosed structural information and our functional analysis, we discussed the receptor recognition and antigenic features of SARS-CoV-2 and SARS-CoV.

The crystal and cro-EM structures of both the SARS-CoV-2- and SARS-CoV-hACE2 complexes revealed that the overall binding modes were quite similar, although the amino acids in the RBMs were quite different. However, how the variable parts of the RBMs in SARS-CoV-2 and SARS-CoV affect receptor recognition had not been well illustrated. In this study, we demonstrated that, six single-amino acid substitutions in SARS-CoV-2 RBD resulted in the loss of favourable interactions with hACE2, namely, N501, Q498, E484, T470, K452 and R439. We also demonstrated that, 5 single amino acid substitutions in SARS-CoV-2 RBD enhanced SARS-CoV-2 RBD-hACE2 binding activity, namely, P499, Q493, F486, A475 and L455. These findings, together with the results of other substitution mutations, confirmed the hypothesis of other published structural articles. Our work provides evidence for the convergent evolution of the SARS-CoV-2 and SARS CoV RBDs and reveals a good example of a functional compensatory evolution mechanism.^24,25^

Remarkably, our data indicated that six substitution mutations in the SARS-CoV-2 RBD, N439/R426, L452/K439, T470/N457, E484/P470, Q498/Y484 and N501/T487, led to the acquisition of enhanced binding affinity for hACE2, which provides clues for monitoring the increased infectibility of natural S protein mutations during the transmission of the virus. The difference in the RBM amino acid sequence raises a new question: Are the protective antigenic sites in the RBD different among SARS-CoVs? We found that the antigenicity antigenic sites of the RBD were distinct in the 2 viruses. We tested a panel of neutralizing mAbs targeting the SARS-CoV RBD, and only one of these antibodies, named CR3022, was able to recognize the SARS-CoV-2 RBD, but it had no neutralizing activity. Notably, the IC_50_ of CR3022 for neutralizing the SARS-CoV pseudo-typed virus was much higher than that of the other four antibodies tested. The latest report from Meng Yuan et al.^13^ revealed the crystal structure of the SARS-CoV-2 RBD in complex with CR3022 and showed that the conserved epitope centred on the S protein did not overlap with the hACE2-binding RBM interface. We also tested a human neutralizing antibody against SARS-CoV-2, named HA001 and purchased from Shanghai Sanyou Biopharma, that showed high-binding affinity for and neutralizing activity against SARS-CoV-2 but no cross-reactivity with SARS-CoV.

We identified Y484 of the SARS-CoV S protein as the key amino acid recognized by SARS-CoV-specific mAbs m396 and 80R. This amino acid was also important for SARS-CoV S-hACE2 binding. Considering that Y484 in SARS-CoV S protein was substituted with Q498 corresponding to SARS-CoV-2 S, this residue may be one of the key amino acids that contributes to the antigenic variation. The speculated epitopes of HA001 was identified as two hACE2 contacting amino acids, A475 and F486, in the SARS-CoV-2 RBM region, which may be new sites for neutralizing antibody binding. Using ELISAs, we also demonstrated that the antisera from mice immunized with mammalian cells expressed recombinant RBDs of SARS-CoV and SARS CoV-2 showed high binding affinity for and neutralizing activity against the respective homologous virus, while the cross-binding and neutralizing activity was much weaker. These results indicated that the RBD domain is a good immunogen to induce clade-specific neutralizing antibodies for disrupting virus-receptor engagement. Regarding the possibility of inducing cross-neutralizing antibodies by immunizing mice with the SARS-CoV RBD, several studies have indicated that natural infection with SARS-CoV or SARS-CoV-2 and immunization of animals with the SARS-CoV RBD induced very limited cross-neutralizing S protein-targeting antibody responses,^26,27^ which is consistent with our observation.

Overall, this study provided clues for developing intervention strategies against SARS-CoV-2. First, although it was not easy to induce cross-protective antibodies, the RBD in SARS-CoV-2 was a potential antigen that could induce abundant neutralizing antibodies against SARS-CoV-2, potentially making it a good candidate for developing subunit vaccines. Second, we demonstrated that SARS-CoV-2 RBM-specific neutralizing mAbs prevented SARS-CoV-2 infection by blocking hACE2 interactions and hence are promising passive antibody-based agents in the absence of an effective prophylactic vaccine. Moreover, the identification of SARS-CoV-2 and SARS-CoV residues important for ACE2 and neutralizing antibody recognition sheds light on the pathogenicity and immune escape mechanisms of SARS-CoV-2.
